# stGuide advances label transfer in spatial transcriptomics through attention-based supervised graph representation learning

**DOI:** 10.3389/fgene.2025.1566675

**Published:** 2025-05-22

**Authors:** Yupeng Xu, Hao Dai, Jinwang Feng, Keren Xu, Qiu Wang, Pingting Gao, Chunman Zuo

**Affiliations:** ^1^ School of Computer Science and Technology, Donghua University, Shanghai, China; ^2^ Key Laboratory of Systems Biology, Shanghai Institute of Biochemistry and Cell Biology, Center for Excellence in Molecular Cell Science, Chinese Academy of Sciences, Shanghai, China; ^3^ College of Computer and Information Science, Chongqing Normal University, Chongqing, China; ^4^ Department of Oncology, Shanghai Medical College, Fudan University, Shanghai, China; ^5^ Guangdong Institute of Intelligence Science and Technology, Zhuhai, China; ^6^ Shanghai Collaborative Innovation Center of Endoscopy, Endoscopy Center and Endoscopy Research Institute Zhongshan Hospital, Fudan University, Shanghai, China; ^7^ School of Life Sciences, Sun Yat-sen University, Guangzhou, China

**Keywords:** spatial transcriptomics, attention-based transfer learning, graph learning, batch effects, label transfer

## Abstract

The growing availability of spatial transcriptomics data offers key resources for annotating query datasets using reference datasets. However, batch effects, unbalanced reference annotations, and tissue heterogeneity pose significant challenges to alignment analysis. Here, we present stGuide, an attention-based supervised graph learning model designed for cross-slice alignment and efficient label transfer from reference to query datasets. stGuide leverages supervised representations guided by reference annotations to map query slices into a shared embedding space using an attention-based mechanism. It then assigns spot-level labels by incorporating information from the nearest neighbors in the learned representation. Using human dorsolateral prefrontal cortex and breast cancer datasets, stGuide demonstrates its capabilities by (i) producing category-guided, low-dimensional features with well-mixed slices; (ii) transferring labels effectively across heterogeneous tissues; and (iii) uncovering relationships between clusters. Comparisons with state-of-the-art methods demonstrate that stGuide consistently outperforms existing approaches, positioning it as a robust and versatile tool for spatial transcriptomics analysis.

## Introduction

Spatial transcriptomics (ST), which provides spatial molecular profiling, has been widely employed to unravel the complex architecture and cellular mechanisms of tissues, particularly in tumors (Arora et al., 2023; Zuo et al., 2022; Zuo et al., 2024). With the expanding repository of research knowledge from ST data across various tissues (Marx, 2021; Wu et al., 2022; Andersson et al., 2021; Wu et al., 2023), a pressing computational challenge emerges: can we utilize suitable reference datasets to annotate new query datasets, thus reducing the need for manual intervention? Mapping a query dataset to a shared embedded reference atlas often faces challenges from batch effects caused by variations in experimental protocols, and inter-tissue heterogeneity.

Several computational approaches have been developed for label transfer from large-scale single-cell RNA sequencing (scRNA-seq) datasets (Lotfollahi et al., 2022; Kang et al., 2021; Hao et al., 2021; Song et al., 2021; Deng et al., 2023). Yet, these approaches often neglect the spatial context, which is crucial for a comprehensive understanding of tissue heterogeneity (Brbić et al., 2022). Recently, a few methods tailored for label transfer in ST data have emerged. Notably, Seurat (Hao et al., 2021), which employs an anchor-based strategy, can be adapted for ST data analysis but was primarily designed for scRNA-seq data. Similarly, STELLAR (Brbić et al., 2022) leverages a graph convolutional neural network to capture spatial and molecular similarities in cell representations, using an adaptive margin mechanism to regulate learning speed. However, it faces challenges with class imbalance, leading to suboptimal predictions for cell categories with few cells.

Transfer learning (TL) models have emerged as a powerful framework for integrating multi-source scRNA-seq data (Hu et al., 2020; Xu K. et al., 2024). Among these, attention transfer, a key TL technique, facilitates the transfer of knowledge from a reference dataset (teacher) to a query dataset (student) in a low-dimensional representation, thereby enhancing the query dataset’s performance (Zuo et al., 2022; Tan et al., 2022; Tjandra et al., 2017; Gong et al., 2013). This approach is particularly well-suited for addressing challenges in the integrative analysis of ST data. In this context, cell representations are learned through supervised models using the reference data. Knowledge is then transferred to the query data by aligning these supervised representations with the unsupervised representation obtained from the joint analysis of both reference and query datasets. This ensures robust cross-dataset integration and accurate label transfer.

Here, we propose stGuide, a model designed for cross-slice integration and alignment, enabling efficient and accurate label transfer for query datasets. To address the challenges of unbalanced categories in reference datasets, stGuide utilizes supervised representations derived from reference annotations to guide the unsupervised representations of both reference and query datasets through attention transfer. Through this approach, stGuide model (i) generates categories-guided, low-dimensional features with evenly mixed slices; (ii) effectively transfers labels from reference to query datasets across diverse heterogeneous tissues; and (iii) infers the relationships between clusters. Through comparative experiments, we show that stGuide outperforms existing methods, providing its robustness and versatility in spatial transcriptomics analysis.

## Results

### Overview of stGuide

**FIGURE 1:**
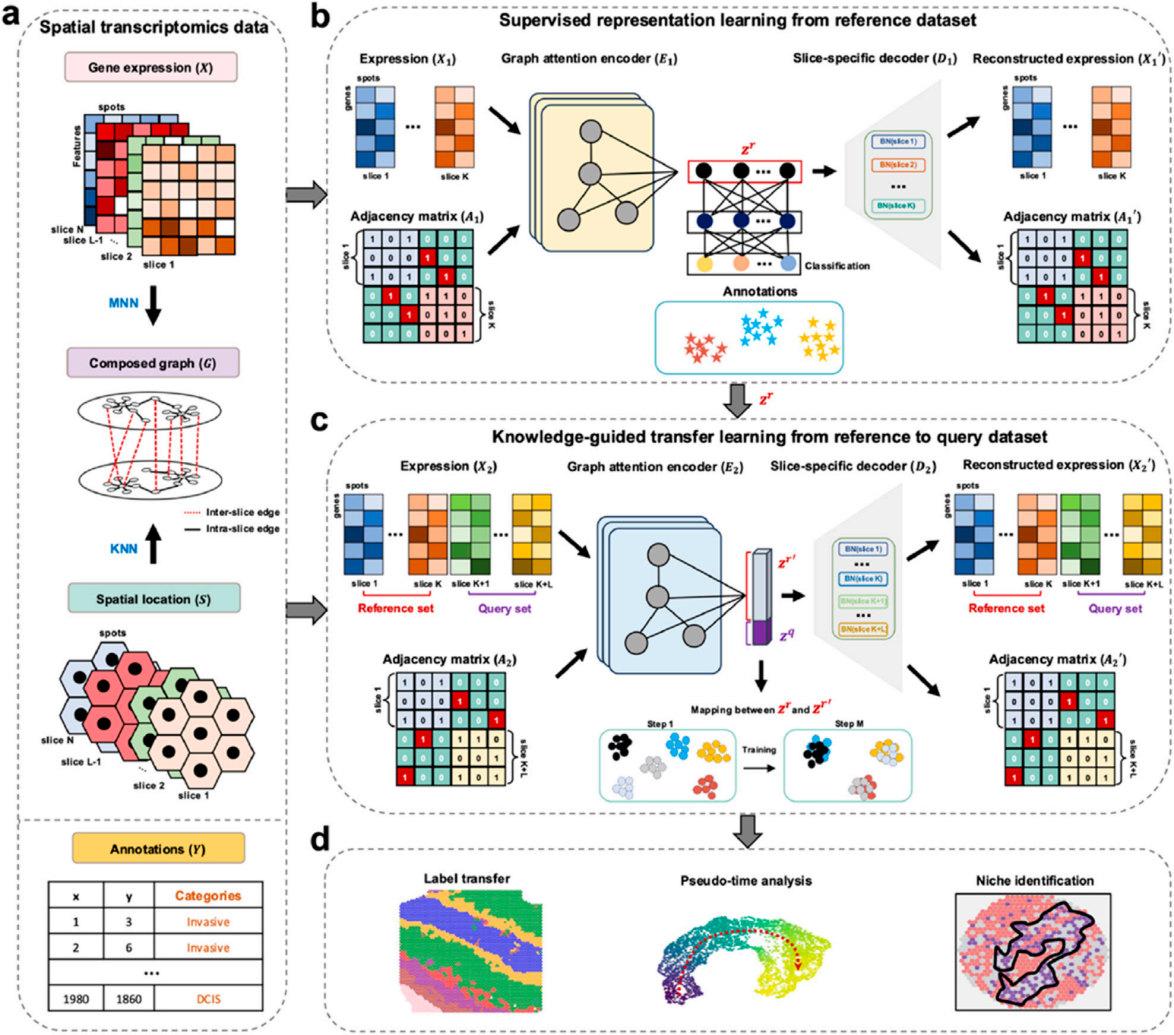
Overview of stGuide. **(a)** Given multiple ST datasets with three-layer profiles: gene expression ( X ) in both query and reference, spatial locations ( S ), and annotations in the reference data ( Y ), stGuide annotates query spot labels based on the reference annotations. A composed graph is constructed using k -nearest neighbors (KNN) for spatial locations and mutual nearest neighbors (MNN) for transcriptomics within and across slices. **(b)** stGuide extracts reference spot representations ( $z^{r}$ ) by aggregating information from spatially and transcriptomic similar spots, guided by spot annotations. **(c)** stGuide learns shared representations for the reference ( ${z^{r}}^{\prime }$ ) and query ( $z^{q}$ ) datasets by aggregating messages from spatially and transcriptomic similar spots, aligning ${z^{r}}^{\prime }$ with the feature space of $z^{r}$ to enable label transfer. **(d)** The representations of query ( $z^{q}$ ) and reference ( $z^{r}$ ) datasets are used for label transfer and pseudo-time analysis.

stGuide introduces an attention-transfer-based supervised graph representation learning model to map query dataset onto reference dataset, enabling label transfer and establishing the relations between different clusters (Figures 1a–d). To address class unbalance, stGuide transfers knowledge from supervised reference representations to unsupervised joint representations of both reference and query slices, ensuring balanced and accurate label propagation.

In the supervised learning module of the reference slices (Figure 1b), stGuide learns representations by capturing spatial and transcriptomics similarities both within and across slices, supervised by known annotations. stGuide employs a graph attention encoder (GAE) to transform $X_{1}$ and $A_{1}$ (the composed graph $G_{1}$ ) into $z^{r}$ , where $z^{r}$ represents batch-corrected features that capture the biological variations inherent in the annotations. The composed graph is constructed by integrating spatial nearest neighbors within each slice and feature-similar neighbors across slices. In the transferring learning module (Figure 1c), stGuide learns cell representations for both reference ( ${z^{r}}^{\prime }$ ) and query slices ( $z^{q}$ ) by capturing spatial and transcriptomics similarities within and across slices. The distance between the supervised reference representations ( $z^{r}$ ) and ${z^{r}}^{\prime }$ is minimized using attention transfer as an additional loss. Subsequently, the labels of query slices are transferred by identifying their k -nearest neighbors in the reference slices, based on the similarity between ${z^{r}}^{\prime }$ and $z^{q}$ . Additionally, the diffusion pseudotime (DPT) algorithm (Haghverdi et al., 2016) is applied to infer pseudo-time relations between clusters within the learned representations (Figure 1d).

### stGuide facilitates label transfer across slices, tissues, and different label quantities

To comprehensively assess the performance of stGuide, we analyzed 12 human dorsolateral prefrontal cortex (DLPFC) slices from three donors, obtained using the 10× Visium platform (Maynard et al., 2021). Each slice was annotated with four or six layers and white matter (WM), serving as the ground truth for evaluating label transfer accuracy. We compared stGuide against Seurat and STELLAR, using accuracy, adjusted rand index (ARI) (Zuo and Chen, 2020), and normalized mutual information (NMI) (Zuo et al., 2021) to evaluate the performance of label transfer between the ground truth and transferred labels. We used four slices 151673-151676 from one donor to train stGuide, Seurat, and STELLAR and then transferred labels to one slice from two independent donors (using slices 151,509 and 151,672 as examples), as well as to multiple slices (151,507-151510) simultaneously.

**FIGURE 2:**
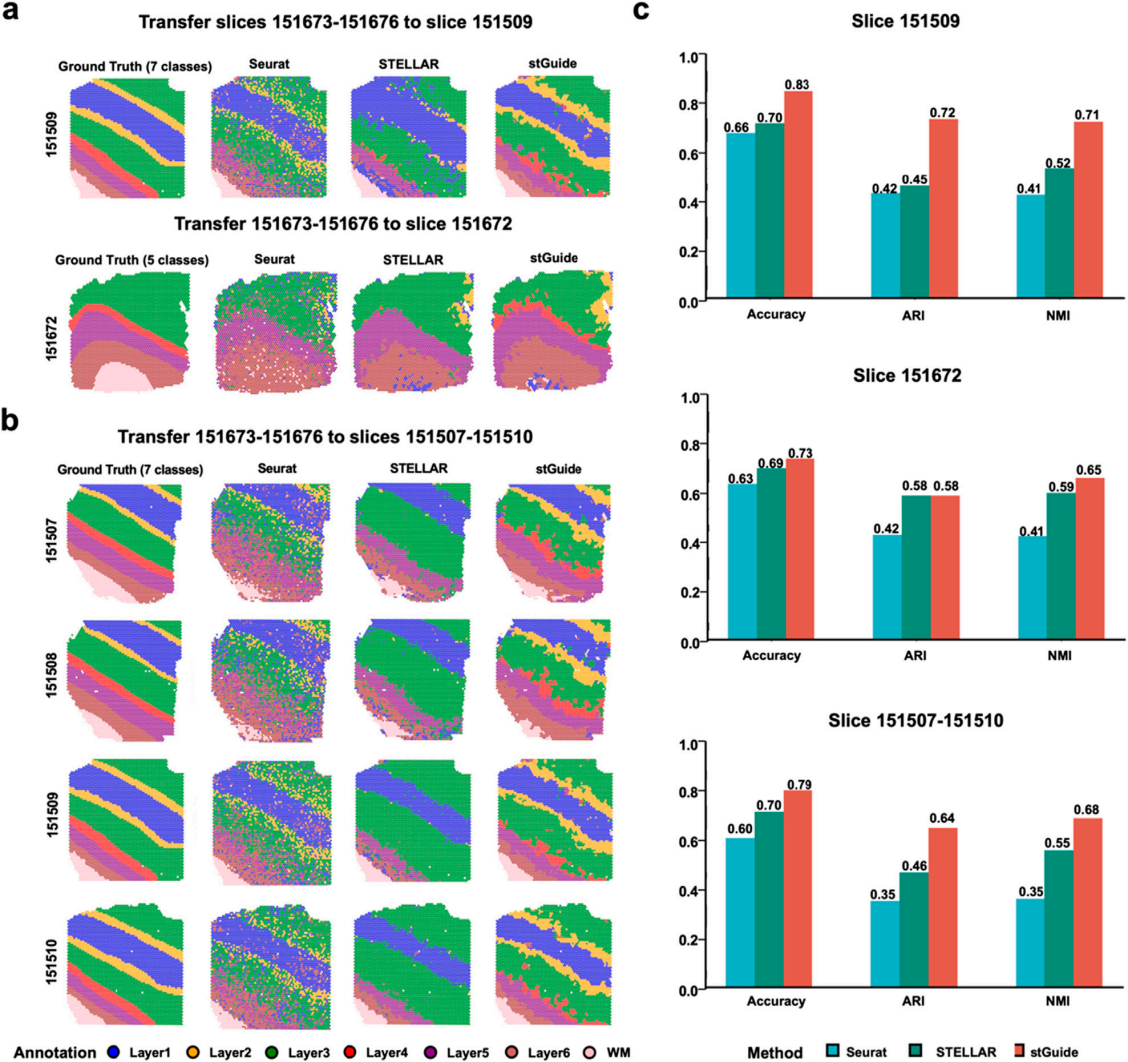
stGuide enables label transfer across slices, tissues, and varying numbers in the human DLPFC dataset. **(a,b)** Four slices (151673-151676) from one donor, annotated with seven layers, were used to train Seurat, STELLAR, and stGuide. The trained models were then applied to transfer labels to slices from independent donors, 151509 and 151672 **(a)**, as well as multiple slices (151507-151510) **(b)**. **(c)** Bar plot showing accuracy, ARI, and NMI for label transfer by the three methods (Seurat, STELLAR, and stGuide) compared to the ground truth on slices from independent donors, 151509 and 151672 **(a)**, as well as multiple slices (151507-151510) **(b)**.

In summary (Figures 2a–c), we found that (1) the labels transferred by stGuide are consistently more accurate than those transferred by Seurat and STELLAR, regardless of whether the transfer was applied to a single slice, multiple slices, or datasets with varying numbers of category labels. This indicates stGuide’s superior generalizability and adaptability across different data configurations; (2) the predicted labels by stGuide show a higher level of concordance with ground truth, particularly in distinguishing layers 2 and 4, as evidenced by achieving the highest ARI and NMI scores; and (3) in the specific case of label transfer on slice 151672, which was annotated with WM and layers 3-6, both stGuide and STELLAR are able to identify layer 2 within the annotated region of layer 3. This outcome not only aligns with our previous findings (Zuo et al., 2024) but also highlights stGuide’s ability to discern subtle distinctions within complex tissue structures, further validating its efficacy in label transfer tasks.

Overall, stGuide effectively transfers labels from the reference tissue to the query tissue by transferring knowledge within the low-dimensional representation space.

### stGuide infers pseudo-time analysis

**FIGURE 3:**
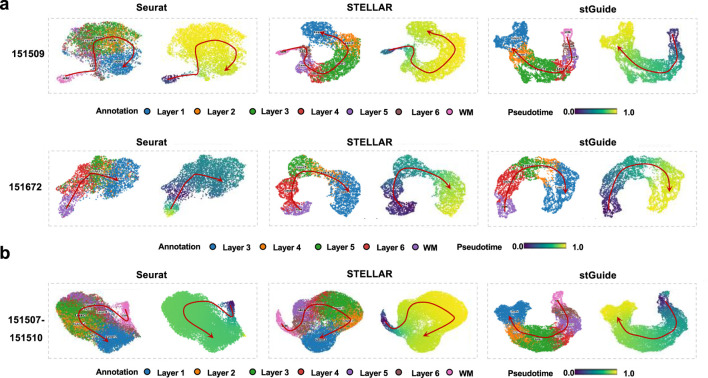
stGuide infers pseudo-time of different layers in the human dorsolateral prefrontal cortex. **(a)** Scatter plot of the two-dimensional UMAP extracted from the representations by Seurat, STELLAR, and stGuide, on slices 151509 and 151672. **(b)** Scatter plot of the two-dimensional UMAP extracted from the representations by Seurat, STELLAR, and stGuide, on multiple slices (151507-151510). For each method of **(a,b)**, the colors of the left and right panels indicate different layers and pseudo-time. Noted that the spatial adjacency and chronological order among these layers are WM → Layer6 → Layer5 → Layer4 → Layer3 → Layer2 → Layer1 (Zuo et al., 2022; Maynard et al., 2021).

One interesting feature of stGuide is its ability to elucidate the relationships between different clusters. To explore this, we applied the DPT algorithm (Haghverdi et al., 2016) to infer pseudo-time trajectories within the low-dimensional features generated by Seurat, STELLAR, and stGuide. Upon comparison, we observed that the pseudo-time inferred by stGuide across different layers, following the progression WM → Layer6 → Layer5 → Layer4 → Layer3 → Layer2 → Layer1, aligns more well with the trajectory of chronological order (Zuo et al., 2022; Maynard et al., 2021) than those inferred by Seurat and STELLAR. This consistency is evident across various scenarios, including single-slice analyses, multiple-slice integrations, and datasets with different numbers of category labels (Figures 3a,b).

In summary, stGuide demonstrates robust performance in capturing the developmental hierarchy of tissue layers, while also maintaining temporal coherence within the spatial transcriptomic landscape, thereby ensuring accurate and consistent label transfer across various datasets and conditions.

### stGuide transfers annotation across cancer slices

We further demonstrated the ability of stGuide to transfer labels across two cancer slices, BAS1 and BAS2, derived from the same heterogeneous breast cancer tissue and publicly available from 10X Genomics. These slices were annotated into 13 tumor regions (Zuo et al., 2024). Using BAS1 for training, we compared the label transfer performance of Seurat, STELLAR, and stGuide on BAS2.

**FIGURE 4:**
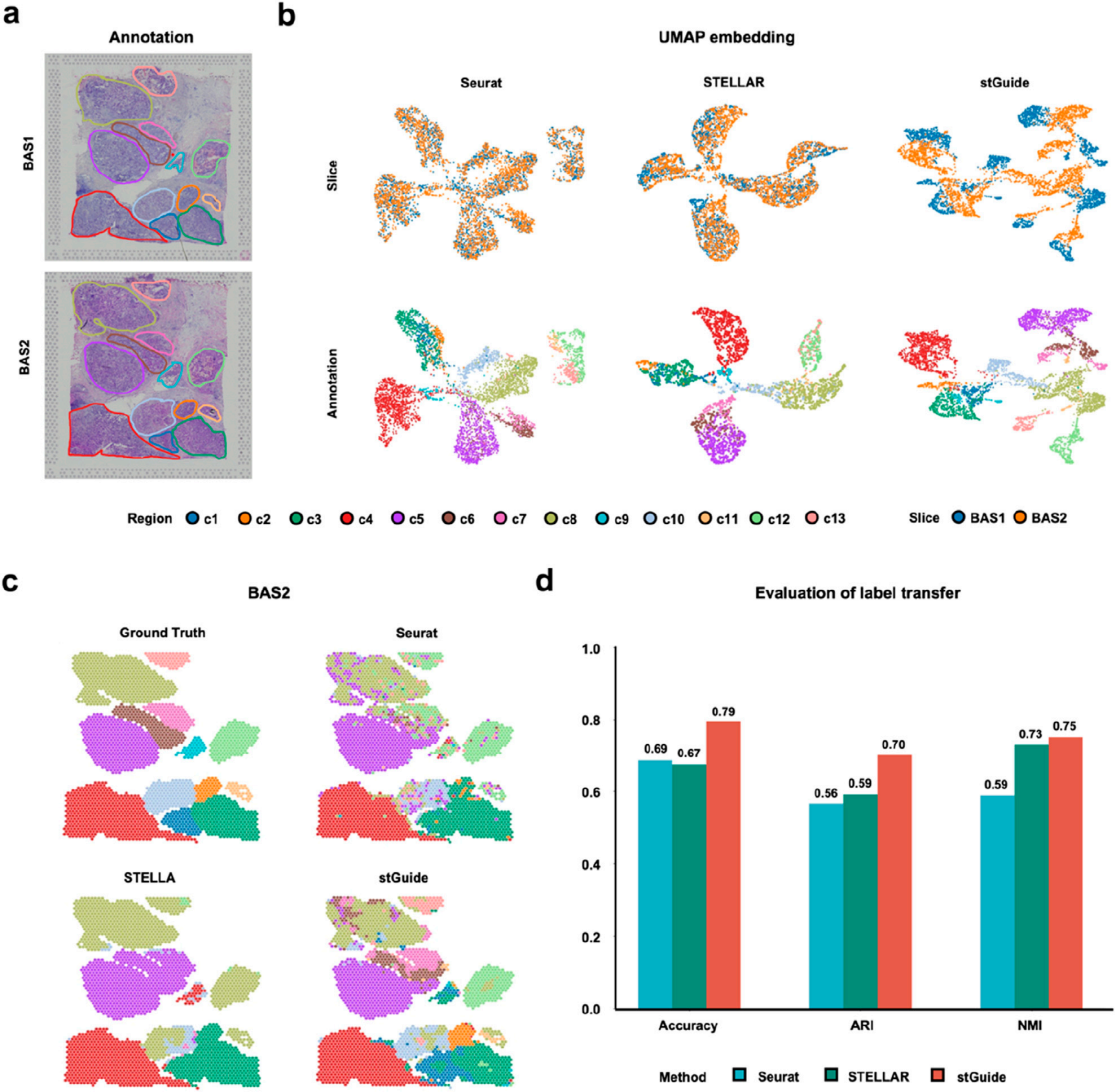
stGuide enables label transfer across heterogeneous slices in the human Luminal B breast cancer sample. **(a)** Spatial plot showing the 13 tumor regions in the BAS1 and BAS2 slices. **(b)** UMAP embeddings of latent features generated by Seurat, STELLAR, and stGuide, with the top panels colored by slices and bottom panels colored by tumor regions. **(c)** Spatial plots illustrating label transfer results from Seurat, STELLAR, and stGuide, compared against manual annotations. **(d)** Bar plot displaying the accuracy, ARI, and NMI for label transfer achieved by the three methods (Seurat, STELLAR, and stGuide) relative to the manual annotation.

By comparison, we found that (1) stGuide accurately predicts labels for ∼80% of spots across the 13 regions in BAS2, outperforming Seurat (∼70%) and STELLAR (∼70%). Moreover, stGuide successfully identifies all 13 regions, whereas STELLAR misclassified regions such as c11, c12, and c13 as c8, as well as c6 and c7 were misidentified as c5. Additionally, regions c1 and c2 were misclassified as c3. Seurat performed worse, failing to detect regions c1, c2, c6, c7, c9, and c11 (Figures 4a–d); and (2) stGuide outperforms both Seurat and STELLAR, achieving an ARI of 0.70 and an NMI of 0.75. Specifically, its NMI exceeds that of STELLAR and Seurat by 0.02 and 0.16, respectively, while its ARI is higher by 0.11 and 0.14. The poor performance of Seurat and STELLAR in the breast cancer datasets is likely due to the following: Seurat relies only on gene expression data without integrating spatial context, limiting its ability to capture tumor heterogeneity. STELLAR struggles with class imbalance, resulting in suboptimal predictions for cell categories with fewer cells.

### stGuide identifies novel cell states missed by competing methods

**FIGURE 5:**
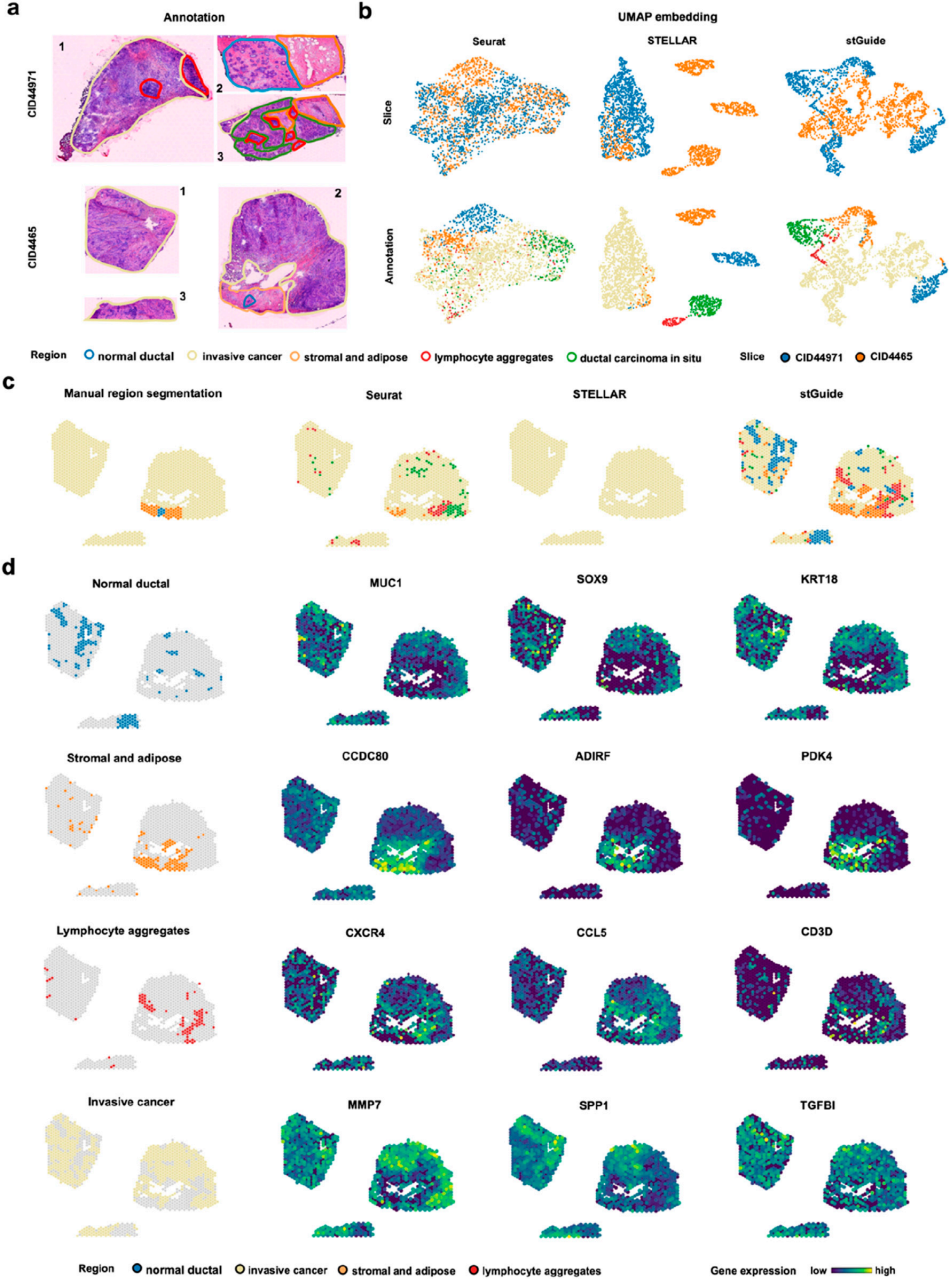
stGuide enables label transfer across human triple-negative breast cancer patients (CID44971 and CID4465). **(a)** H&E-stained plots showing the annotations of five histological regions on slices CID44971 and CID4465, with each color representing one region. **(b)** UMAP embeddings generated by Seurat, STELLAR, and stGuide. The top panel is colored by slice, while the bottom panel is colored by histological annotations. **(d)** Spatial clusters identified by Seurat, STELLAR, and stGuide, with annotations provided for comparison. **(d)** Spatial distribution of gene expression levels for marker gene in different regions: normal ductal region (*MUC1*, *SOX9*, and *KRT18*), stromal and adipose regions (*CCDC80*, *ADIRF*, and *PDK4*), lymphocyte aggregations (*CXCR4*, *CCL5*, and *CD3D*), and IC region (*MMP7*, *SPP1*, and *TGFBI*).

To further clarify that stGuide can establish relationships between tumor samples across heterogeneous patients, we applied it to analyze two triple-negative breast cancer slices, CID44971 and CID4465 ^26^. The slices were annotated into five histological regions: normal ductal, invasive cancer (IC), stromal and adipose, lymphocyte aggregations, and ductal carcinoma *in situ* (DCIS) (Figure 5a). Slice CID44971 was used to train Seurat, STELLAR, and stGuide, and the labels from slice CID44971 were transferred to slice CID4465 for comparison.

Through comparison, we observed that (i) the features learned by Seurat and stGuide exhibited better mixing across slices compared to STELLAR (Figure 5b); and (ii) Seurat misclassified some spots in the IC region as DCIS, while STELLAR inaccurately predicts the entire slice as IC. In contrast, stGuide successfully identifies distinct regions, such as stromal and adipose, and even uncovered regions unannotated in CID4465, including lymphocyte aggregations, which were not identified in prior research (Wu et al., 2021), (Figure 5c).

To verify these findings, we calculated the differential genes for each region on slice CID44971 and examined their expression levels on slice CID4465. The results showed that marker genes of the normal ductal region (*MUC1*, *SOX9*, and *KRT18*), stromal and adipose regions (*CCDC80*, *ADIRF*, and *PDK4*), lymphocyte aggregations (*CXCR4*, *CCL5*, and *CD3D*), and IC region (*MMP7*, *SPP1*, and *TGFBI*) were over-expressed in the corresponding regions predicted by stGuide (Figure 5d). These findings highlighted stGuide’s ability to accurately detect and delineate both annotated and previously annotated regions across heterogeneous tumor samples.

## Discussion

stGuide is a graph-based transfer learning model designed for label transfer and trajectory inference in ST, effectively addressing challenges including batch effects, category imbalance, and inter-tissue heterogeneity. Specifically, stGuide (1) leverages a shared graph encoder to map reference dataset into a category-informed embedding, while utilizing slice-specific decoders to reconstruct graph and feature profiles, where the embedding is supervised by the reference annotations; and (2) maps both query and reference datasets into a shared embedding using a similar graph structure, with slice-specific decoders for graph and feature reconstruction. The reference embedding is learned through attention-guided supervision from reference annotations. Through two-step training, stGuide enables the generation of category-guided, low-dimensional features with evenly mixed slices, effective label transfer across heterogeneous tissues, and the identification of relationships between clusters.

Benchmark comparisons on the human DLPFC dataset revealed that stGuide consistently outperforms other methods in label transfer accuracy, regardless of whether the transfer involved a single slice, multiple slices, or datasets with varying numbers of category labels. Additionally, the pseudo-time inferred by DPT from stGuide’s representations aligned more closely with known chronological trajectories, highlighting its ability to capture biologically meaningful features. Evaluations on human breast cancer samples demonstrated stGuide’s unique strengths in cross-slice label transfer and uncovering niches in the query dataset based on histological annotations (Wu et al., 2021). Moreover, we further demonstrated the effectiveness of stGuide on datasets with cellular and subcellular resolution, demonstrating its adaptability across varying spatial scales. stGuide achieved superior performance compared to existing methods, further confirming its robustness across spatial resolutions (Supplementary Figure S3).

In future studies, we aim to expand our work in two key directions: (1) leveraging the wealth of cell-state information in histological images (Zuo et al., 2022) and the rapid advancements in foundational models for computational biology and pathology (Lu et al., 2024; Xu H. et al., 2024; Cui et al., 2024), we will develop sophisticated algorithms to seamlessly integrate histological images into cross-slice spatial transcriptomics analysis. This integration will be achieved by using deep learning models (such as convolutional neural networks or vision Transformers) to extract local image features corresponding to each spot, while employing attention mechanisms (such as multi-head attention or graph attention networks) to dynamically fuse image features with transcriptomic features. The resulting joint representation will enhance the resolution and interpretability of cell-state mapping, particularly compensating for the limited spatial resolution of transcriptomic data in complex tissues (e.g., tumors or brain tissues); (2) as diverse datasets expand (Xu Z. et al., 2024; Zhang et al., 2021; Zhang et al., 2024) and graph models advance (Mao et al., 2022), we will optimize stGuide to efficiently handle large-scale datasets through three approaches: improving the computational efficiency of graph attention mechanisms, introducing distributed computing frameworks, and developing incremental learning methods. These enhancements will improve the tool’s generalizability and adaptability across various biological and pathological contexts, ultimately establishing stGuide as a core platform for spatial transcriptomics analysis. This advancement will provide powerful technical support for understanding complex biological systems and advancing precision medicine research.

## Methods

### stGuide model

stGuide integrates multi-slice gene expression data ( $X=\left(x_{1},\ldots ,x_{K+L}\right),x_{i}\in R^{m\times n_{i}}$ ), spatial location data ( $S{=\left(s_{1},\ldots ,s_{K+L}\right),s}_{i}\in R^{n_{i}\times 2}$ ), and reference slices annotations ( $Y=\left(y_{1},\ldots ,y_{K}\right),y_{i}=\left\{y_{i,1},\ldots ,y_{i,n_{i}}\right\}\in R^{n_{i}\times 1},y_{i,j}\in \left\{1,\ldots ,K_{a}\right\}$ ) for cross-slice alignment and label transfer using an attention-based supervised graph model. Here, K and L are the number of reference and query slices, and m , $n_{i}$ , and $K_{a}$ represent the common features, spots in the ith slice, and categories in the ith slice, respectively. Specifically, stGuide learns supervised representation from reference slices using known annotations, maps query slices into the same embedding space via an attention map, and predicts spot labels by leveraging nearest neighbors in representation (Figures 1a–d).

### Supervised representation learning from the reference dataset

stGuide extracts spot features ( $z^{r}\in R^{d\times n}$ ) from the reference dataset by integrating spatial and transcriptomic information from similar spots both within individual slices and across multiple slices, guided by annotations, where d and n are dimension size and the number of spots, respectively (Figure 1b). Specifically,

#### Construction of composed graph

We constructed a composed graph ( $G_{1}=\left(V_{1},E_{1}\right)$ ) to create links between spots across multi-slices, utilizing gene expression and spatial location data. Intra-slice edges were established by calculating the Euclidean distance between spots, maintaining an average of five nearest neighbors using the k -nearest neighbors. Inter-slice edges, connecting spot pairs from different slices, were identified as mutual nearest neighbors (MNN) based on feature similarity, with a default of five nearest neighbors, employing the MNN method (Haghverdi et al., 2018) (Figure 1a).

#### Encoding features by supervised graph learning model

We learned supervised features $z^{r}$ through a shared GAE by combining gene expression data of the reference dataset with an adjacency matrix $A_{1}$ (representing the composed graph $G_{1}$ ). To ensure accurate representation, we employed K slice-specific BatchNorm decoders to reconstruct each slice’s gene expression data and adjacency matrix. This process was guided by annotations Y , ensuring that spots belonging to the same groups were jointly embedded together, thereby enhancing the biological coherence of the representations.

(i)Encoder: the specific encoder structure of GAE consists of multiple stacked multi-head graph attention layers (GAT). Each layer is defined as follows (Equations 1, 2):

$$h_{i}^{l+1}=ELU\left(\frac{1}{Q}\sum_{q=1}^{Q}\sum_{j\in N_{i}}a_{ij}^{q}W^{q}h_{j}^{l}\right)$$ (1)

$$a_{ij}^{q}=\frac{\exp\left(LeakyReLU\left({\left(a^{q}\right)}^{T}\left[W^{q}h_{i}^{l}\|W^{q}h_{j}^{l}\right]\right)\right)}{\sum_{o\in N_{i}}\exp\left(LeakyReLU\left({\left(a^{q}\right)}^{T}\left[W^{q}h_{i}^{l}\|W^{q}h_{o}^{l}\right]\right)\right)}$$ (2)

where Q denotes the number of attention heads, with a default value of 3, determined based on our experiment results (Supplementary Figure S2). The multi-head attention mechanism enables model performance by capturing stable and expressive spot representations from both spatially and omics-similar neighbors, thereby improving label transfer accuracy; $N_{i}$ represents the neighboring nodes of the spot i , while $h_{j}^{l}$ indicates the input features of the node j in the lth GAT layer; $W^{q}$ is the linear transformation weight matrix for input features in the qth attention head; and $a_{ij}^{q}$ refers to the normalized attention coefficients computed by the qth attention head using SoftMax activation. The encoder consists of two layers of GAT layers, with the first- and second-layers having dimensions of 512 and 10, respectively;

(ii) Decoder of gene expression: the one-layer linear decoder specific to the ith slice, along with BatchNorm, is used to reconstruct ith gene expression data ( ${X_{i}}^{\prime }$ ) from the latent feature ${z_{i}}^{r}$ (Chang et al., 2022) (Equations 3, 4):

$$X_{i}^{\prime }=\gamma_{i}\times \frac{h_{ij}-\mu_{i}}{\sqrt{\sigma_{i}^{2}+\epsilon }}+\beta_{i}$$ (3)

$$h_{ij}=W{z_{i}}^{r}+b$$ (4)

where the dimension of $h_{i\cdot }$ matches $X_{i}$ , $\mu_{i}$ , and $\sigma_{i}^{2}$ are the mean and variance of spots in the ith slice, $\gamma_{i}$ and $\beta_{i}$ handle slice-specific scaling and shifting parameters, and ϵ is a constant. The loss function is summarized as follows (Equation 5):

$${L_{G}}^{r}=\frac{1}{n}\sum_{i=1}^{n}{\left\|{X_{i}}^{\prime }-X_{i}\right\|}_{2}$$ (5)

(iii) Decoder of adjacency matrix: an inner product between the embedding ${z_{i}}^{r}$ is used to reconstruct the adjacency matrix ( ${A_{1}}^{\prime }=sigmoid\left({z_{i}}^{r}{{z_{i}}^{r}}^{T}\right)$ ), and the corresponding loss function is summarized as follows (Equation 6):

$${L_{A}}^{r}=\frac{1}{n\times n}\sum_{i=1}^{n}\sum_{j=1}^{n}\left({a_{ij}}^{r}\times \log\left({{a_{ij}}^{r}}^{\prime }\right)+\left(1-{a_{ij}}^{r}\right)\times \log\left(1-{{a_{ij}}^{r}}^{\prime }\right)\right)$$ (6)

where ${a_{ij}}^{r}$ and ${{a_{ij}}^{r}}^{\prime }$ represent the elements in the ith row and jth column of the adjacency matrices $A_{1}$ and $A_{1}^{\prime }$ , respectively.

(iv) Classifier: To incorporate group information into low-dimensional features, we extended the GAE model to predict spot classes $Y^{\prime }=softmax\left(Wz^{r}\right)$ , guided by the annotation. The loss function is described as follows (Equation 7):

$$L_{c}=\frac{1}{S}\sum_{i=1}^{S}\left(-\sum_{i=1}^{K_{a}}y_{i}\log\left({y_{i}}^{\prime }\right)\right)$$ (7)

where S denotes the number of spots in the reference dataset, $K_{a}$ represents the number of classes, and $y_{i}$ and ${y_{i}}^{\prime }$ are the label vectors for spot $v_{i}$ from the ground truth and predicted outputs, respectively.

In summary, the loss function of the supervised graph learning module is defined as (Equation 8):

$$L_{pre}={L_{A}}^{r}+\alpha {L_{G}}^{r}+\beta L_{c}$$ (8)

where α and β are used to control the weight of each term, both set to 10, as this configuration yielded the best performance in our evaluation experiments (Supplementary Figure S1).

### Knowledge-guided transfer learning from reference to query dataset

stGuide employs a shared graph encoder to learn spot features for the query dataset ( $z^{q}\in R^{d\times u}$ ) and the reference dataset ( ${z^{r}}^{\prime }\in R^{d\times n}$ ), ensuring ${z^{r}}^{\prime }$ is aligned with the supervised features $z^{r}$ using an attention map as additional loss, thereby enabling label transfer within the representation space, where u represents the number of spots in the query dataset (Figure 1c). Specifically,

(i) Unsupervised graph modeling: We used the same structure for the unsupervised graph encoder and decoder in the supervised representation learning module to learn spot features. This process involved integrating gene expression data from both the reference and query datasets using an adjacency matrix $A_{2}$ (the composed graph $G_{2}$ ). To reconstruct each slice’s gene expression data and adjacency matrix, we employed K+L slice-specific BatchNorm decoders. As a result, the loss functions for reconstructing gene expression and the adjacency matrix are consistent with Formulas 5, 6, referred to as ${L_{G}}^{q+r}$ and ${L_{A}}^{q+r}$ , respectively.
(ii) Knowledge-guided transfer learning: To map ${z^{r}}^{\prime }$ into the same feature space as $z^{r}$ , we introduced a penalty loss function to minimize the difference between ${z^{r}}^{\prime }$ and $z^{r}$ for the corresponding spots in the reference dataset (Equation 9):

$$L_{TL}=\frac{1}{K}\sum_{i=1}^{K}{\left\|{z_{i}}^{r}-{{z_{i}}^{r}}^{\prime }\right\|}_{2}$$ (9)

where K represents the number of slices in the reference dataset.

Overall, the loss function of the knowledge-guided transfer learning module is summarized as (Equation 10):

$$L_{q+r}={L_{A}}^{q+r}+{\eta {L_{G}}^{q+r}+\theta L}_{TL}$$ (10)

where η and θ are used to control the weight of each term, both set to 10, as this configuration yielded the best performance in our evaluation experiments (Supplementary Figure S1).

After model training, the learned features for the query ( $z^{q}$ ) and reference datasets ( ${z^{r}}^{\prime }$ ) are used for downstream analysis (Figure 1d). For each query spot, stGuide computes the cosine similarity between its embedding and all reference embeddings. The label of the reference spot with the highest similarity—indicating the closest spot in the latent space—is assigned to the query spot. This approach ensures interpretable and biologically meaningful label transfer based on the learned embedding space (Figure 1d).

### Datasets and preprocessing

In this study, we analyzed publicly available ST datasets, including human brain, breast cancer, and mouse brain samples. Specifically, (1) the human DLPFC dataset contains 12 slices from three independent donors, each with four adjacent slices. Each slice was manually annotated into four (or six layers) and white matter (WM) to evaluate label prediction accuracy; (2) the human Luminal B breast cancer dataset includes two slices (BAS1 and BAS2) from the same tissue, containing 3,798 and 3,987 spots, respectively. Pathologists annotated 13 tumor regions on each slice by analyzing H&E images; (3) the human triple-negative breast cancer (TNBC) dataset contains two slices (CID44971 and CID4465) from different patients, with 1,162 and 1,211 spots, respectively; (4) the mouse hypothalamus dataset includes two slices (MERFISH_Data26 and MERFISH_Data27), containing 5,557 and 5,926 spots, respectively. Based on annotation information, the dataset was divided into 8 regions; (5) the mouse medial prefrontal cortex dataset includes two slices (STARmap_31 and STARmap_32), with 1,049 and 1,053 spots, respectively. Based on annotation information, the dataset was divided into 4 regions (Li et al., 2022).

For each slice, we followed the standard scanpy workflow (Wolf et al., 2018), including normalization and log transformation of raw gene expression. Subsequently, we selected the top 5,000 highly variable genes (HVGs) per slice. The intersection of HVGs across all slices was considered as common genes, and their horizontal concatenation across all spots from multiple slices formed the input data X .

### Visualization

We employed the “tl.umap” function from the scanpy package (Wolf et al., 2018) to map the shared low-dimensional features into a two-dimensional UMAP space, visualized the spatial embeddings of different spatial domains using the “pl.umap” function, and inferred the pseudo-time through “tl.dpt” function. We also used the “pl.spatial” function to visualize the clustering and gene expression distribution at the spatial level for each slice.

### Evaluation of label transfer

We evaluated label transfer performance using three metrics: accuracy, adjusted rand index (Zuo and Chen, 2020), and normalized mutual information (Zuo et al., 2021). Accuracy reflects the proportion of correctly predicted spots in the query dataset and is defined as follows (Equations 11, 12):

$$P\left(i\right)=\left\{\begin{array}{c} 1,y_{i}^{\prime }=y_{i}\\ 0,y_{i}^{\prime }\neq y_{i} \end{array}\right.$$ (11)

$$Accuracy=\frac{\sum_{1}^{n}P\left(i\right)}{n}$$ (12)

where $y_{i}$ and $y_{i}^{\prime }$ represent the true and predicted label information for each spot, respectively.

ARI measures pairwise consistency between predicted and true labels, defined as follows (Equations 13, 14):

$$RI=\frac{TP+TN}{TP+FP+TN+FN}$$ (13)

$$ARI=\frac{RI-E\left[RI\right]}{\max\left(RI\right)-E\left[RI\right]}$$ (14)

where TP represents pairs in the same cluster in both true and predicted labels, TN denotes pairs in different clusters in both labelings, FP indicates pairs that are split in true labels but merged in predicted labels, FN refers to pairs that are merged in true labels but split in predicted labels, and $E\left[RI\right]$ is the expected value of RI under random clustering.

NMI quantifies mutual dependence between the prediction and true label based on information theory (Equation 15):

$$NMI=\frac{2\cdot I\left(Y,Y^{\prime }\right)}{H\left(Y\right)+H\left(Y^{\prime }\right)}$$ (15)

where $I\left(Y,Y^{\prime }\right)$ is the mutual information between true labels Y and predicted labels $Y^{\prime }$ , and $H\left(\cdot \right)$ denotes entropy. The NMI metric ranges from 0 to 1, with values closer to 1 indicating stronger statistical dependence between predicted labels and true labels.

## Data Availability

The original contributions presented in the study are included in the article/Supplementary Material, further inquiries can be directed to the corresponding authors.
