# Supplementary material for: stGuide advances label transfer in spatial transcriptomics through attention-based supervised graph representation learning
Source: Front Genet. 2025 May 22;16:1566675. doi: 10.3389/fgene.2025.1566675 (PMC12137301; doi:10.3389/fgene.2025.1566675)
Supplement: Supplementary file 1 [file Supplementaryfile1.docx]

**Supplementary Figure Legends**

**Supplementary Figure 1.** Line plot showing the performance metrics (Accuracy, ARI, and NMI) of stGuide for label transfer tasks from slices 151673-151676 to slice 151509 in the DLPFC dataset under different hyperparameters. The hyperparameter was evaluated at values of 1, 5, 10, 15, and 20 to assess its impact on model performance.

**Supplementary Figure 2.** Line plot displaying the performance metrics (Accuracy, ARI, and NMI) of stGuide for label transfer tasks from slices 151673-151676 to slice 151509 in the DLPFC dataset under varying multi-head attention settings. The number of attention heads was tested at values of ranging from 1 to 6 to evaluate its impact on model performance.

**Supplementary Figure 3. stGuide enables label transfer across high-resolution slices. a-b** Spatial plots showing the 8 annotated regions in MERFISH (Data26 and Data27 slices; subcellular resolution) and 4 regions in STARmap (Data31 and Data 32 slices; cellular resolution). **c-d** Spatial plots comparing label transfer results from Seurat, STELLAR, and stGuide against manual annotations in MERFISH (**c**) and STARmap (**d**) datasets. **e-f** Bar plots displaying accuracy, ARI, and NMI for label transfer performance across the three methods (Seurat, STELLAR, and stGuide) on both MERFISH (**e**) and STARmap (**f**) datasets.
